# The effect of hemorrhagic shock and intraosseous adrenaline injection on the delivery of a subsequently administered drug - an experimental study

**DOI:** 10.1186/s13049-018-0569-z

**Published:** 2019-03-08

**Authors:** Mats Eriksson, Anders Larsson, Miklós Lipcsey, Gunnar Strandberg

**Affiliations:** 1 Section of Anesthesiology & Intensive Care, Department of Surgical Sciences, Uppsala University Hospital, Uppsala, Sweden; 2 Section of Clinical Chemistry, Department of Medical Sciences, Uppsala University Hospital, Uppsala, Sweden; 3 Hedenstierna Laboratory, Department of Surgical Sciences, Uppsala University Hospital, Uppsala, Sweden

**Keywords:** Adrenaline, Cardiac arrest, CPR, Intraosseous, Pig, Shock

## Abstract

**Background:**

Intraosseous (IO) access is a recommended method when venous access cannot be rapidly established in an emergency. Experimental data suggest that major hemorrhage and catecholamine administration both reduce bone marrow blood flow. We studied the uptake of gentamicin as a tracer substance administered IO following adrenaline administration in hemorrhagic shock and in cardiac arrest.

**Methods:**

Twenty anesthetized pigs underwent hemorrhage corresponding to 50% of the blood volume. They then received injections of either; adrenaline IO (n = 5), saline IO n = 5), adrenaline IO during cardiac arrest and cardiopulmonary resuscitation (CPR, n = 5), or intravenous adrenaline. The injections were followed by an injection of gentamicin by the same route. Doses and volumes were equivalent among the groups. In all animals, mixed venous antibiotic concentrations were analyzed at 5, 15 and 30 min after administration.

**Results:**

Mean (SD) plasma gentamicin concentrations (mg x L^− 1^) at 5 min were 26.4 (2.3) in the group with previous IO adrenaline administration, 26.6 (4.5) in the IO saline group, 31. 2 (12) in the IO adrenaline + CPR group and 23 (4.5) in the IV group. Concentrations in the CPR group were significantly higher than the others.

**Conclusions:**

No impairment of drug uptake with IO administration after recent IO adrenaline exposure was demonstrable in this shock model.

## Background

Intraosseous (IO) cannulation is indicated when intravenous access is not quickly established in a medical emergency and evidence indicates that this method is relatively straightforward with a high success rate and a low frequency of serious complications in emergency medicine and prehospital settings [1–5]. Among other situations, it is frequently used in military and civilian trauma, and in cardiopulmonary resuscitation (CPR) where it is the recommended route for administration of adrenaline and other drugs if venous access is unavailable [6, 7]. There is however experimental evidence indicating that bone marrow blood pressure and flow decreases in hypovolemia and circulation may be substantially reduced both during hemorrhagic shock and following cardiac arrest and systemic catecholamine administration [8, 9]. Therefore, it could be hypothesized that the central delivery of substances administered during hypovolemia and after IO adrenaline administration might be impaired.

We have previously demonstrated a good uptake of gentamicin when administered IO in the tibia in a porcine sepsis model, with a concentration-time curve very similar to that seen after intravenous administration [10].

The aim of the present study was to evaluate drug uptake with IO administration after major hemorrhage and repeated doses of adrenaline, both of which have been demonstrated to reduce bone marrow blood flow and will likely often be relevant factors in situations where IO access is used. Uptake was also studied after IO adrenaline administration during CPR in hypovolemia. Based on previous experience we chose gentamicin as a model substance for drug uptake.

## Methods

### Animals

Twenty healthy pigs with a mean (SD) weight of 25.1 (1.8) kg were included in the study. The animals were treated according to the guidelines of the Swedish Board of Agriculture and the European Convention on Animal Care. The Animal Ethics Committee of Uppsala University, Sweden, approved the experiment (C155/14, date of approval 24-10-2014).

### Anesthesia and preparation

All animals were anesthetized with an injection of 6 mg x kg^− 1^ tilétamin-zolezepam and 2.2 mg x kg^− 1^ xylazin intramuscularly in the neck. General anesthesia was maintained with an infusion of 8 mg x kg^− 1^ x h^− 1^ of pentobarbital mixed with 1.6 mg x kg^− 1^ x h^− 1^ of rocuronium bromide and 0.48 mg x kg^− 1^ x h^− 1^ of morphine.

After bolus doses of 20 mg of morphine and 100 mg of ketamine the animals were tracheotomized and mechanical ventilation was initiated with a Servo I® ventilator (Maquet Critical Care, Solna, Sweden). The animals continuously received 8 mL x kg^− 1^ x h^− 1^ of a solution containing 25 mg x mL^− 1^ of glucose, and 7 mL x kg^− 1^ x h^− 1^ of Ringer’s Acetate. An arterial catheter was placed into a right cervical artery and a central venous catheter was introduced through a right cervical vein into the superior caval vein. A Swan-Ganz catheter was introduced through a right cervical vein into the pulmonary artery for monitoring purposes. In order, not to compromise the cervical circulation, we avoid both the carotids and the internal jugular veins. Instead we catheterize the central vessels through a branch of the thyrocervical trunk and the right external jugular vein. A 15G IO cannula (EZ-IO®, Teleflex corp. Morrisville, NJ) was inserted into the proximal tibia. Correct placement was verified by the needle standing firmly in the bone, by aspiration and by an incision to the bone after finishing the experiment. A 13.5 F catheter was inserted into a left cervical vein. A small vesicotomy was made and a urinary catheter was inserted into the urinary bladder.

### Protocol

Hemorrhagic shock was induced in all animals by removal of 50% of the calculated (67 ml x kg^− 1^) blood volume through the 13.5 F venous catheter followed by resuscitation with the same volume of Ringer Acetate [11].

Animals were randomly divided in 4 groups with *n* = 5/group. Based on data from previous experiments, with an α of 0.05, a sample size of 20 animals was calculated to give an 80% power of detecting a 25% difference in mean concentration between groups, which was considered acceptable in this exploratory study.

In group 1, three bolus doses of adrenaline, 0.01 mg x kg^− 1^ in dilution 1:10000, were administered in the IO catheter with an interval of 4 min in accordance with current CPR practice guideline recommendations [7]. Each dose was followed by a 10 ml 0.9% saline bolus. Immediately after the third adrenaline dose a bolus injection of gentamicin, 7 mg x kg^− 1^, was administered, also followed by a 10 ml 0.9% saline bolus.

Animals in group 2 received, through the IO cannula, three bolus doses of 10 ml 0.9% saline with 4 min interval followed by gentamicin as described above, but no adrenaline.

In group 3, anesthesia was deepened with further injections of ketamine and morphine after which a left sided thoracotomy was performed. After this, cardiac arrest was induced by IV potassium injection and internal heart massage was commenced. This procedure prolonged the preparation phase by approximately 30 min. During CPR, adrenaline and gentamicin was administered IO in a manner identical to that in group 1. Internal heart massage was performed manually by two operators with change over every few minutes upon agreement.

In group 4, adrenaline and gentamicin were administered in the same manner and dose as in group 1, but through an 18G IV cannula sited in an ear vein instead of IO.

CPR operators were not blinded to any experimental aspects but the laboratory performing the gentamicin analyses were blinded to group allocation and mode of administration.

In all animals, blood was sampled from the pulmonary artery catheter at 5, 15 and 30 min after gentamicin administration, after which the experiment was terminated.

### Laboratory analysis

Gentamicin concentrations were analyzed on an Architect Ci8200 analyzer (Abbott Laboratories, Abbott Park, IL) using reagent from the same manufacturer (1P31). The total coefficients of variation (CV) for the gentamicin assay were 1.7% at 3.0 mg x L^− 1^ and 2.2% at 5.5 mg x L^− 1^.

### Data analysis

Plasma gentamicin concentrations were assessed for normality. Concentrations at 5, 15 and 30 min were compared among the groups using one-way ANOVA and Tukey HSD test for post-hoc analysis. The area under the plasma concentration-time curve (AUC) was also calculated for each individual, using the trapezoidal rule, and compared among groups with ANOVA and Tukey HSD. Statistica 13 software (Statsoft Inc. Tulsa, OK) was used for analyses and graphics.

## Results

**Fig. 1:**
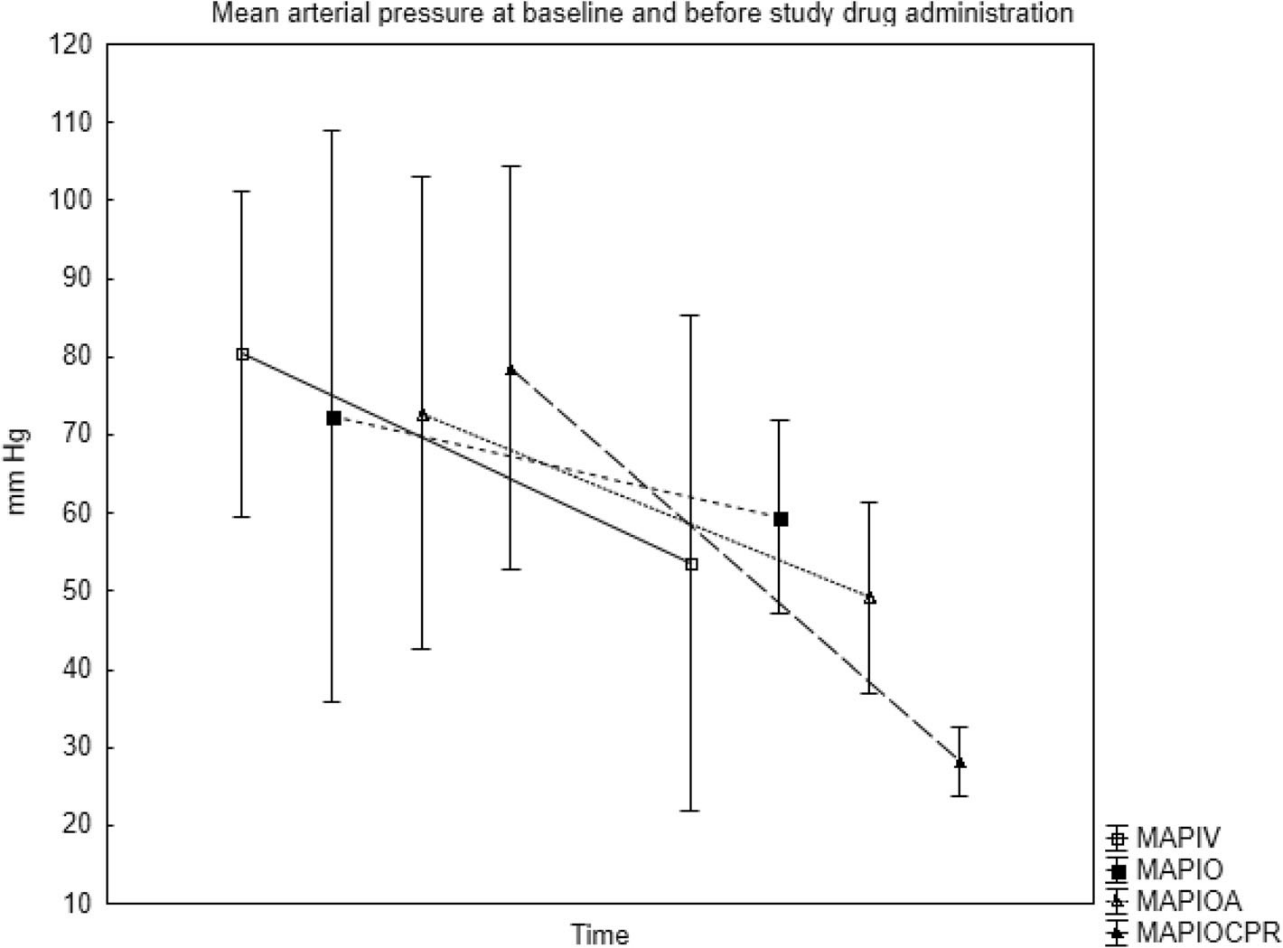
Mean (SD) arterial pressure (MAP) in all groups at baseline (after anesthesia induction) and prior to study drug administration. IO = IO gentamicin, no adrenaline, IOA = IO adrenaline + gentamicin, IV = IV adrenaline + gentamicin, IOCPR = IO adrenaline + gentamicin, cardiopulmonary resuscitation

**Fig. 2:**
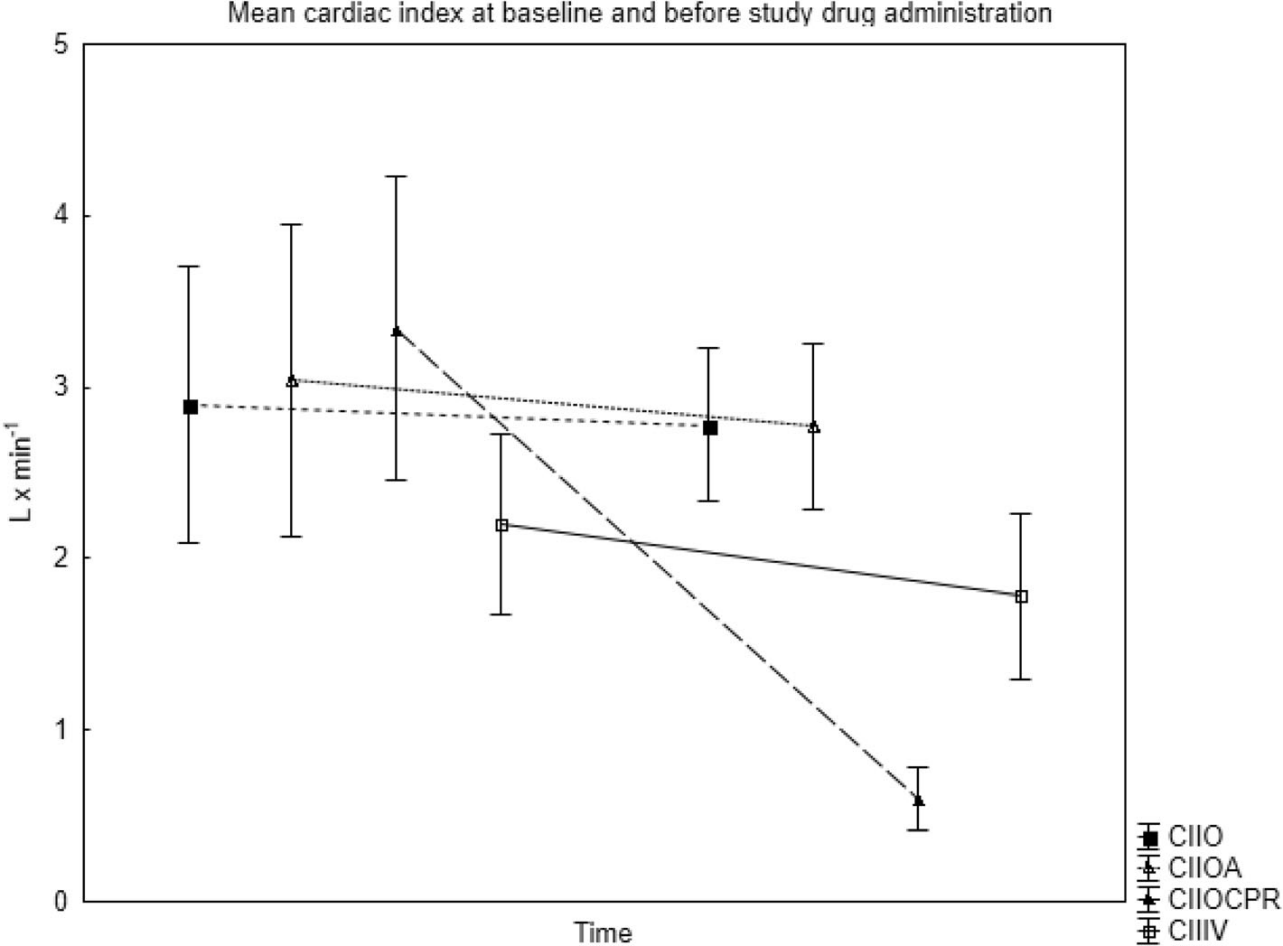
Mean (SD) cardiac Index (CI) in all groups at baseline (after anesthesia induction) and prior to study drug administration. IO = IO gentamicin, no adrenaline, IOA = IO adrenaline + gentamicin, IV = IV adrenaline + gentamicin, IOCPR = IO adrenaline + gentamicin, cardiopulmonary resuscitation

Hemorrhage with partial resuscitation of the animals resulted in a lowered mean arterial pressure while the cardiac index was less affected. During CPR, both parameters were very low. Hemodynamic data at baseline and at the time of drug administration for all groups are presented in Figs. 1 and 2. After IO adrenaline administration, within 10–20 s a marked increase in heart rate and blood pressure was noted in the groups with preserved cardiac activity.

**Fig. 3:**
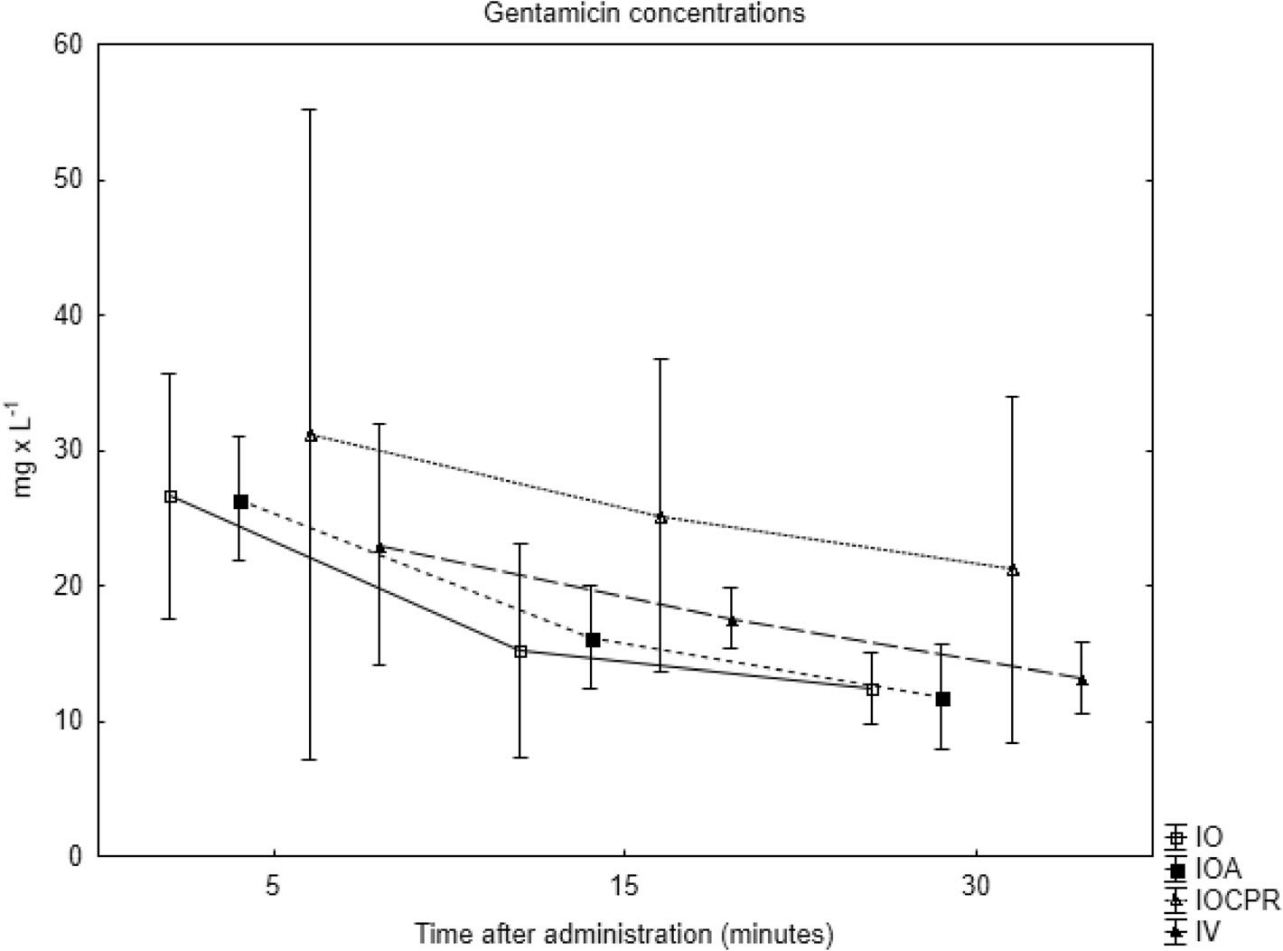
Pulmonary artery gentamicin concentrations at 5, 15 and 30 min after administration for all groups. Data are mean (SD). IO = IO gentamicin, no adrenaline, IOA = IO adrenaline + gentamicin, IV = IV adrenaline + gentamicin, IOCPR = IO adrenaline + gentamicin, cardiopulmonary resuscitation

Mean (SD) gentamicin plasma concentrations at 5, 15 and 30 min for all groups are presented in Fig. 3. Mean (SD) concentrations at 5 min were 26.4 (2.3) mg x L^− 1^ in group 1, 26.6 (4.5) mg x L^− 1^ in group 2, 31.2 (12) mg x L^− 1^ in group 3 and 23 (4.5) mg x L^− 1^ in group 4. At 5 min, concentrations were not significantly different among the groups, but at 15 and 30 min they were higher in group 3 compared with the others (*p* < 0.05).

The AUC (mg x h x L^− 1^) was also significantly larger in group 3; mean (SD) 10.5 (2.9), while the other groups were similar; IO 6.9 (1.2), IO Adrenaline 7.1 (0.7), IV 7.2 (0.4).

## Discussion

In this experimental study, intraosseous administration yielded clinically relevant concentrations of gentamicin after administration of either adrenaline or saline. Concentrations were equivalent to those after intravenous administration.

The rationale for using a porcine model of hemorrhagic shock is mainly due to the suitable size of the pig, which allowed us to bleed the animals, perform CPR, monitor, administer drugs IO and IV, and to easily take blood samples. Although the proximal humerus may be preferred for IO access [4], we used the proximal tibial route, in order to reduce the risk of interference between the IO needle and practical management of open-chest cardiac massage. Tracheotomy, instead of oro-tracheal intubation, was performed, since we, in previous, long-term (> 30 h), experiments have had several animals that developed pneumonia. Our assumption is that the source of these infections was contaminated debris in the mouth of the animals. Hence, we tracheotomize the animals due to hygiene reasons. Since this is a model of hemorrhagic shock induced cardiac arrest, internal heart massage was performed. In hypovolemic cardiac arrest, such as in trauma, internal heart massage is not unusual, wherefore we chose this setup.

IO access is commonly used in clinical emergencies e.g. cardiopulmonary resuscitation and shock of hypovolemic or other origin. In this setting it is likely that the IO cannula will be used for the administration of adrenaline or other catecholamines. In this situation, it is important to know if the uptake of subsequently administered drugs is adequate. Examples of this could be antiarrhythmics in a CPR scenario, antibiotics in a case of septic shock or hemostatics in trauma. In this experiment, gentamicin was studied as a model substance because of a previously demonstrated good uptake when administered IO. In the hypovolemic animals with preserved cardiac activity, we found no significant difference between plasma concentrations of gentamicin administered IO with or without previous adrenaline injections, even though the antibiotic was not given with a large volume of fluid. Gentamicin levels were also not significantly different in samples taken after IO and IV administration during similar hemodynamic conditions. This indicates that the experimentally demonstrated impairment of bone marrow circulation after catecholamine exposure and hypovolemia may not pose a serious clinical problem in this context. In the group where adrenaline and gentamicin were administered during CPR in hypovolemic animals, plasma concentrations were significantly higher than in the other groups. Since samples were taken relatively early after administration, when redistribution predominates over elimination [12], this is more likely an effect of a reduction in cardiac output than of decreased excretion. However, this still indicates that uptake from the bone marrow has occurred.

This study has several limitations. Although the staff performing the laboratory analyses were blinded to group allocation, the people performing CPR were not, which may be considered a limitation. Also, the thoracotomy and induction of cardiac arrest delayed the start of the experimental procedure by approximately 30 min, compared with the other groups. However, we do not believe that this limitation significantly affects our results. It should be noted that this is an experimental study performed in animals, and that the results therefore may not be directly translated to human conditions. Further, the number of subjects is limited and the study will not detect minor differences. In an emergency however, it may be sufficient to know that adequate central concentrations may quickly be reached after an IO bolus injection following repeated adrenaline administration. In this study, gentamicin was used as a model substance. We have, however, no reason to believe that previous adrenaline administration would have a different effect on the uptake of other substances.

## Conclusions

In this model of hemorrhagic shock, IO administration of adrenaline did not impair the subsequent uptake of gentamicin administered through the same cannula. The plasma concentrations of gentamicin were equivalent to those after IV administration.
